# Address before the Onondaga County Homœopathic Medical Society

**Published:** 1887-11

**Authors:** William A. Hawley


### ADDRESS BEFORE THE ONONDAGA COUNTY HOMŒOPATHIC MEDICAL SOCIETY.

WILLIAM A. HAWLEY, M. D.

FELLOW-MEMBERS:—It is simply impossible for me to say to you how earnestly I desire this Society to be a really active, energetic, living organization. To further this end, if it may be, allow me to say some things to you as brethren.

At our last meeting the question was, "Why is this Society not an efficient help to each of its members?" Now I raise another: How shall we make it such a help? Bear with me while I state what seems to me essential to that end. First of all, we need *oneness of motive*—a feeling that we have a cause to sustain which is peculiarly our own, and its support is the first end of all our seeking, inasmuch as it is our common purpose and common end. That cause is Homœopathy, as expressed in the law of cure, "*similia similibus curantur,*" and its corollaries, the *single remedy*, because it is the most like, and there can be but one most like, and the *minimum dose,* since the question always is, How little will cure? instead of, as in the old school, How much will the patient bear? In these days of the rapid multiplication of doctors in medicine, there is a constantly increasing tendency to break away from such a common cause and common motive, and fall into the entirely opposite state of selfish and personal ends, and a spirit not of helpfulness, but of rivalry and jealousy which ever seeks to pull down others for the sake of upbuilding oneself—in short, of falling into just such a spirit of competition as makes the world of trade. No association of physicians can be mutually helpful to its members into which there comes even the slightest trace of this spirit. I say we must have a cause, and it must be, if *real*, indeed, will be, nearer to each of us than any personal ends. That cause is Homœopathy, pure and simple, and cannot be anything else, for if we grant for the moment, which we do not, that the method of the old school has merit, that field is already better cultivated by those wholly devoted to it than it can possibly be by any who adopt two methods which are confessedly directly opposed to each other. By pursuing such a course we not only fail to help each other, but we can but lose our own self-respect and the respect of all intelligent thinking people, and so fail to help ourselves. If we will make this Society a means of growth, we must do it by our earnest study of Homœopathy, its philosophy, and its practical application in the treatment of the sick. Our school has won its position and power simply and only by such study and application of its *law*. This position and power is recognized and practically confessed by the old school. There is no longer active warfare on their part.

Skepticism in all medicine is the rule among them, and their fight against Homœopathy has practically ceased, the only weapon left them being ridicule of infinitesimal doses. Still, for all this, Homœopathy has yet its greatest enemy to overcome. Because of a prevalent materialism which is in the very atmosphere of our time, there is among our school the same skepticism which pervades the old school, and creates the same disbelief of infinitesimal doses and the same uncertainty and fear at the bedside. This naturally results in a tendency to material doses, and at once our self-deceived homœopathist has fallen into an eclecticism more confusing and uncertain than that of the old school. This condition of things is, of course, lamented and opposed by all who see the inside of things, so that, indeed, they *know* that everywhere and always it is the inside and not the outside that is the verity, and that it is the spirit-like (dynamic) force of the drug which is the active agent.

This doubt of the immaterial side of things causes a denial of the truth of the potentization of drugs at last beyond a fixed limit, and a consequent denunciation of those who accept it as extremists, and therefore unsafe and unworthy of public confidence. So, everywhere they decry the true follower of Hahnemann as a high dilutionist, who gives only water medicine. All this is, to say the best of it, wholly a mistaken view of the situation, since if they understood those men they would know that with them all, without exception, the question of dose is an open one, while they hold themselves at liberty to use the whole range of potencies, and do use any of them as they see occasion. Really, therefore, the split in homœopathic ranks is not on the question of potency, but on the question of *conformity to law*—the law of things which requires, if we will cure the sick with drugs, we shall give the one drug that produces in well people symptoms most like those existing in the case to be cured. This law is a positive prohibition of any and all eclecticism. More than that, it renders it *certain that conformity to it must and will prevail.* To talk of choosing the best from both methods sounds very wise and is very taking with the thoughtless public, but did any one ever detect the powers of the universe in doing the same thing in two different ways? To presume that we can do it is to arrogate to ourselves a wisdom that even the Almighty does not possess. I am happy to believe that the force of this truth is being more and more felt by those who have even a weak faith in Homœopathy. More and more they are beginning to see that success as healers can only come through *obedience to law.* Long ago it was discovered that the best is only attained by seeking it, and that in attaining the best we necessarily go through all the good between our present state and the best. Yet so small is our faith in this greatest of truths that we are often tempted to go contrary to our best judgment in the treatment of the sick that we may hold the patient and save our credit. If we yield, what have we gained? Only our own disrespect.

Almost two thousand years ago there lived a man who found out, and said it, that " he that would save his life shall lose it," and all the world finds it so true that we think God said it. Fellow-workers for humanity, let us ponder these things and be true to ourselves, and we cannot be false to law, nor can we fail.

To do this, study your weapons, *our almost omnipotent Materia Medica.* So shall we be helps to each other, while we add honor to our glorious cause, and the world shall be the better for our living in it.

